# Enhancing the Mechanical Properties of Cu–Al–Ni Shape Memory Alloys Locally Reinforced by Alumina through the Powder Bed Fusion Process

**DOI:** 10.3390/ma16113936

**Published:** 2023-05-24

**Authors:** Daniyal Abolhasani, Ha-Neul Kwon, Yong-Han Park, Young-Hoon Moon

**Affiliations:** Center for Innovative Technology on Advanced Forming, School of Mechanical Engineering, Pusan National University, Busan 46241, Republic of Korea; daniyal@pusan.ac.kr (D.A.); qmffn2203@pusan.ac.kr (H.-N.K.); yonghan@pusan.ac.kr (Y.-H.P.)

**Keywords:** shape memory, additive manufacturing, Cu–Al–Ni, local-reinforcement, alumina, fracture mode

## Abstract

A classical problem with Cu-based shape memory alloys (SMAs) is brittle fracture at triple junctions. This alloy possesses a martensite structure at room temperature and usually comprises elongated variants. Previous studies have shown that introducing reinforcement into the matrix can refine grains and break martensite variants. Grain refinement diminishes brittle fracture at triple junctions, whereas breaking the martensite variants can negatively affect the shape memory effect (SME), owing to martensite stabilization. Furthermore, the additive element may coarsen the grains under certain circumstances if the material has a lower thermal conductivity than the matrix, even when a small amount is distributed in the composite. Powder bed fusion is a favorable approach that allows the creation of intricate structures. In this study, Cu–Al–Ni SMA samples were locally reinforced with alumina (Al_2_O_3_), which has excellent biocompatibility and inherent hardness. The reinforcement layer was composed of 0.3 and 0.9 wt% Al_2_O_3_ mixed with a Cu–Al–Ni matrix, deposited around the neutral plane within the built parts. Two different thicknesses of the deposited layers were investigated, revealing that the failure mode during compression was strongly influenced by the thickness and reinforcement content. The optimized failure mode led to an increase in fracture strain, and therefore, a better SME of the sample, which was locally reinforced by 0.3 wt% alumina under a thicker reinforcement layer.

## 1. Introduction

Shape memory alloys (SMAs) have demonstrated their utility in diverse applications ranging from aerospace to everyday life, such as dampers, valves, and hydraulic tube couplings. The damping property in the martensitic state is one of the unique properties of bulk SMAs, wherein recovery occurs without the need for additional force [1].

Functional actuators and sensors can be made from Ni–Ti alloys and Cu-based binary and ternary alloys, such as Cu–Al, Cu–Zn, Cu–Al–Ni, and Cu–Zn–Al alloys [2]. However, for high-temperature applications, Ni–Ti alloys cannot be used because they are low-temperature SMAs. Ni–Ti–X (X = Hf, Zr, Pd), Cu-based alloys, TiTa-based alloys, Co–Ni–Ga alloys, and Ni–Mn–Ga alloys are high-temperature SMAs. Owing to their good corrosion resistances and low manufacturing costs, Cu–Al–Ni SMAs have garnered wide attention from researchers. Coarse grain size, fracture at triple junctions, and brittleness are the most common issues found in the application of Cu–Al–Ni alloys in the required areas [3]. Grain refinement by adding an element and heat treatment have been utilized in order to broaden its applicability [4,5]. The effects of the addition of Nd, boron, Mn, Co, Zr, and Ti on the mechanical properties and the shape memory effect (SME) of Cu–Al–Ni alloys show that significant improvements have been achieved [6,7,8]. The addition of additional elements to the Cu–Al–Ni alloy affects the fabrication cost. However, if the alloy is subjected to high temperatures, grain segregation due to elemental reactions is amplified. Furthermore, the additive element may coarsen the grains because of its lower thermal conductivity than that of the matrix [9]. Three-dimensional printing can significantly tailor the microstructure and functional properties of Cu-based SMAs via element concentration, the generation of nanoprecipitates, and changes in endothermic–exothermic enthalpies [10,11,12]. However, by tailoring the size and distribution of elements and phases, simultaneous improvement in the mechanical properties and shape memory properties of SMAs still remain as challenges [13].

In the current study, to improve the damping characteristics of a Cu–Al–Ni alloy subjected to a compressive environment, a local reinforcement approach around the neutral plane of samples fabricated using laser three-dimensional (3D) printing was proposed. This approach focuses on the thickness of the locally deposited reinforcement layer, reinforcement content, and fracture mode of the samples. At the center area of the printed sample, a layer comprising alumina-reinforced Cu–Al–Ni with no specific pattern was deposited at two different thicknesses, representing the low- and high-reinforcement layers. Alumina (Al_2_O_3_) reinforcement was selected because of its excellent biocompatibility. The aim of this study was to provide a new understanding of samples produced via the 3D printing process with a locally reinforced region around the neutral plane. The fracture mode under a compressive force was then examined in order to correlate it with the shape recovery ratio of the fabricated samples used as dampers at high temperatures.

## 2. Experimental

**Figure 1:**
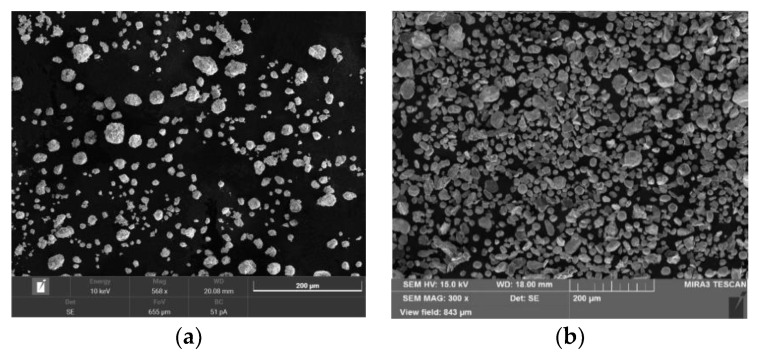

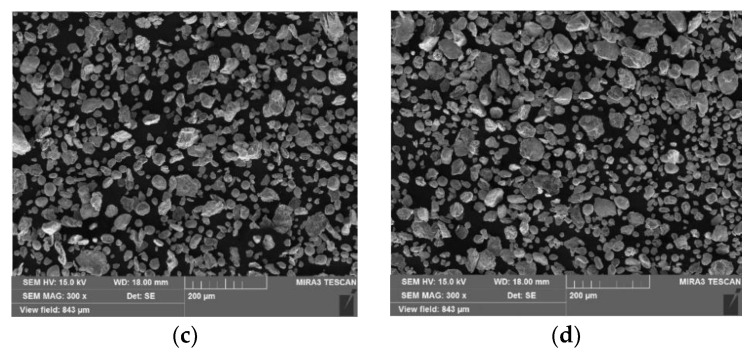
SEM images of: (**a**) Al_2_O_3_. (**b**) Cu–Al–Ni. (**c**) Al_2_O_3_ 0.3 wt%-reinforced Cu–Al–Ni. (**d**) Al_2_O_3_-0.9 wt%-reinforced Cu–Al–Ni.

**Figure 2:**
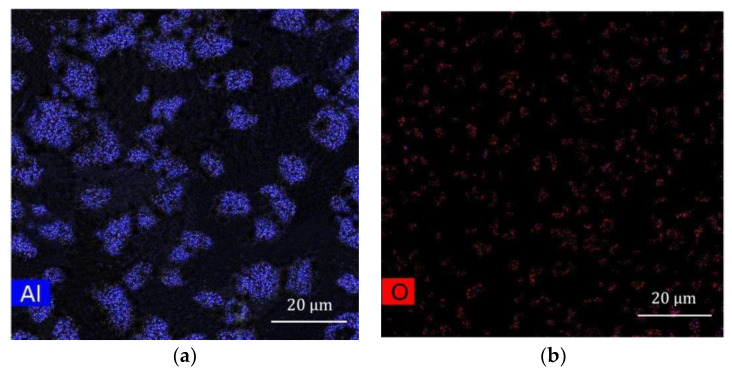
EDX elemental maps of: (**a**) Al, and, (**b**) O, in Al_2_O_3_ 0.9 wt%-reinforced Cu–Al–Ni powder mixture.

The initial powders of Cu, Al, and Ni with particle sizes of 30, 30, and 40 µm, respectively, and 99.9 wt% purity, and Al_2_O_3_ particles with an average particle size of 5 µm, were purchased from AVENTION Co., Ltd. (Jacksonville, FL, USA). It is already known that the alloy exhibits a memory effect when the Al content is in the range of 10–15 wt%. Powder mixtures of 82 wt% Cu, 14 wt% Al, and 4 wt% Ni were mechanically alloyed. The weight percentage of Al_2_O_3_ was determined to be 0.3 and 0.9 wt% in order to not have a significant effect on density, and it was replaced by Cu in the mixtures [9]. A rotational speed of 200 rpm with a ball-to-powder weight ratio of 5:1 was applied to the mixture for 5 h during the ball milling. The average sizes of the particles were 20–30 µm and 60–70 µm. The morphology of Al_2_O_3_ is shown in Figure 1a, and the resultant powder mixtures are shown in Figure 1b–d. Figure 2 confirms the uniform distribution of Al_2_O_3_ particles in Al_2_O_3_ 0.9 wt%-reinforced Cu–Al–Ni sample using EDX elemental maps of Al and O that show no signs of agglomeration within the powder stock.

**Figure 3:**
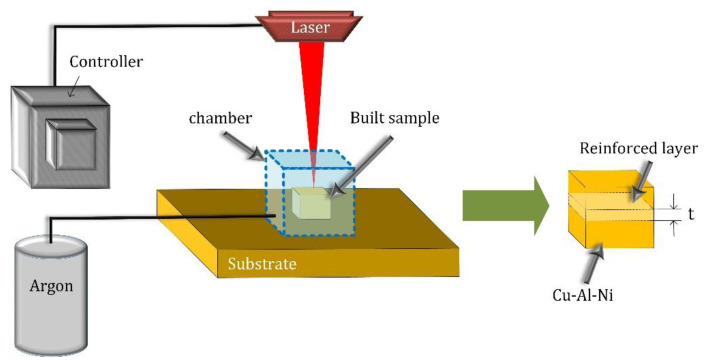
Schematic of the laser process and a built sample.

**Figure 4:**
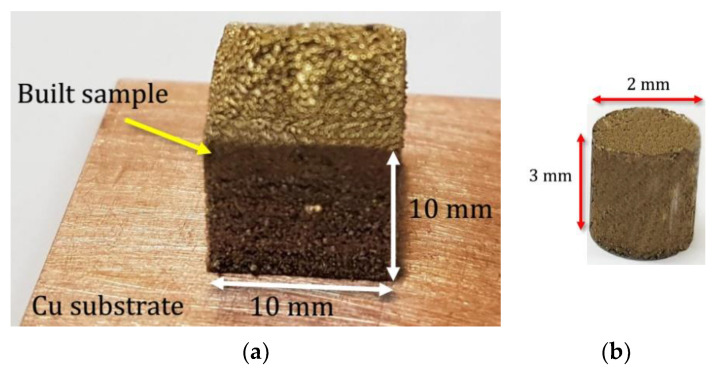
(**a**) Printed sample. (**b**) A compressive sample extracted from the fabricated part.

The laser was an nLight quasi continuous wave (nLight-QCW) fiber laser system with a maximum power of 500 W. The laser spot diameter and layer thickness were 80 and 50 µm, respectively. The scanning strategy between the layers was an x–y alternation. The reinforced layer was deposited at the center of the printed samples. Figure 3 illustrates the building process under an Ar atmosphere and the proposed method for fabricating locally reinforced samples. In this figure, t is the reinforced layer thickness with a magnitude of 100 and 500 µm. The optimum conditions for the laser process were determined based on the density measurements. A laser power of 300 W and a scan speed of 400 mm/s were identified for the Cu–Al–Ni samples, which were also applied to reinforced samples [9]. Figure 4a shows the built sample. The compressive samples were extracted from the built parts, as shown, for instance, in Figure 4b.

Prior to the microstructural evolution, the samples were polished and a solution of 30 mL distilled water, 20 mL HCl, and 15 mL HNO_3_ was used to etch the samples for 3–4 min. A TESCAN MIRA3 FE-SEM (TESCAN, Czech Republic) system equipped with an EDS analyzer was used for scanning electron microscopy (SEM) examination. A DSC25 system was utilized to measure the transformation temperatures. To calculate the SME, the height of the compressive samples was measured after wire cutting (L_0_), unloading (L_1_), and heating/cooling (L_2_) when samples were heated to Ap for 10 min, which is the temperature at which each sample falls within its average austenite temperature range. Thus, the recovered strain, or ε_SME_, was (L_2_ − L_1_)/L_0_ × 100. The pre-strain (loading strain) was set to two different values of 5 and 10%.

## 3. Results and Discussion

### 3.1. Microstructure

The phase transition temperatures are listed in Table 1. The decrease in Al content caused by the reaction with Al_2_O_3_, resulting in the formation of precipitates and Al depletion, was the cause of the increase in the austenite and martensite temperatures with the addition of Al_2_O_3_ [9,14].

**Figure 5:**
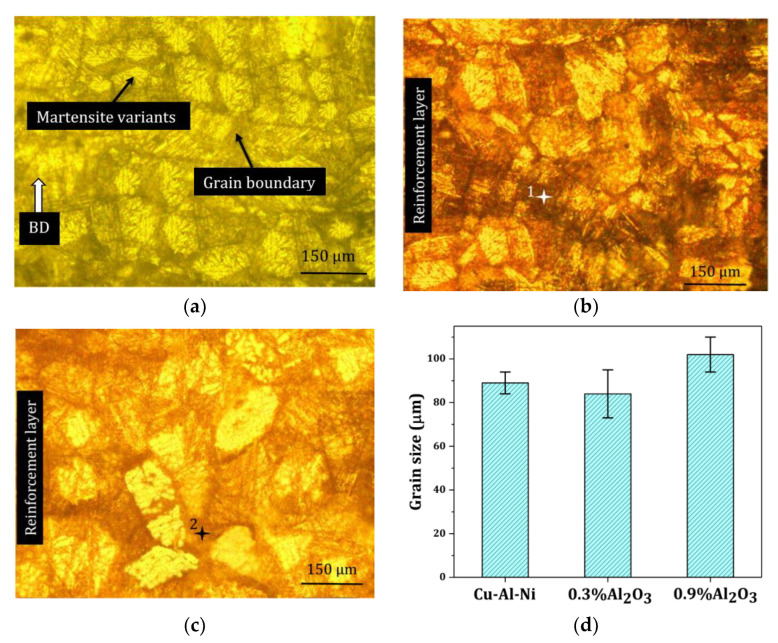
The morphology of the grains obtained from optical microscopy: (**a**) Cu–Al–Ni, (**b**) Cu–Al–Ni-0.3Al_2_O_3_, and (**c**) Cu–Al–Ni-0.9Al_2_O_3_. Two numbered symbols (+) denote the regions for EDS analysis. (**d**) The average values of grain sizes (BD: building direction).

Figure 5a–c shows the mid-layer cross-sections of the metallographic micrographs of Cu–Al–Ni, Cu–Al–Ni-0.3Al_2_O_3_, and Cu–Al–Ni-0.9Al_2_O_3_. The thickness of the reinforced layer was set at 500 µm (10 deposited layers). Coarse grains were observed in all samples. Therefore, the reinforcement particles do not play a significant role in breaking and refining the grains. Even though only a small amount was added to the alloy to prevent the built samples from reaching low cooling rates [15,16], Figure 5d depicts the grain sizes through optical microscopy, showing that with the addition of 0.9 wt% Al_2_O_3_, the grain size increased because of the low thermal conductivity of Al_2_O_3_ versus copper.

**Figure 6:**
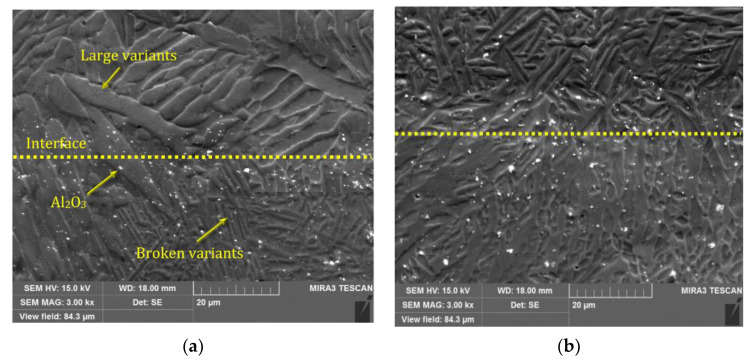
The SEM images of: (**a**) Cu–Al–Ni-0.3Al_2_O_3_, (**b**) Cu–Al–Ni-0.9Al_2_O_3_.

Figure 6 shows the SEM images of the interface between the reinforced layer and the matrix. Broken martensite variants exist in the regions containing reinforcement particles, whereas the variants appear in a larger size in the non-reinforced region. It was concluded that the reinforcement particles could break the martensite variants rather than the grains. With increasing Al_2_O_3_ content, the martensite variants were continuously refined, as shown in Figure 6b. The greater trapping of the reinforcement element inside the matrix variants in Cu–Al–Ni-0.9Al_2_O_3_ was responsible for its variants’ refinement. The lower Marangoni flow caused by the ceramic particles with low thermal conductivity [17,18], in addition to the ceramic’s low sensitivity to grain misorientation, reduced the diffusion and aggregation of Al_2_O_3_ particles at the grain boundaries [19]. This led to a small variation in the grains, as shown in Figure 5d, but a greater variation in size. EDS analysis on spectra 1 and 2 in Figure 5b,c shows an almost constant amount of O in two samples with a low amount of Al, as demonstrated in Table 2. Thus, one can deduce that the increase in Al_2_O_3_ content from 0.3 to 0.9 wt% had no remarkable effect on alumina concentration in the grain boundaries.

According to Figure 6a, both the small variants of 18R β′_1_ martensite and the large plate shape variants of 2H γ′_1_ martensite co-exist at the interface in Cu–Al–Ni-0.3Al_2_O_3_, whereas the small variants exist at the interface of Cu–Al–Ni-0.9Al_2_O_3_ in Figure 6b. This could affect the SME because smaller variants are able to inhibit martensite transformation owing to greater entanglement [20].

**Figure 7:**
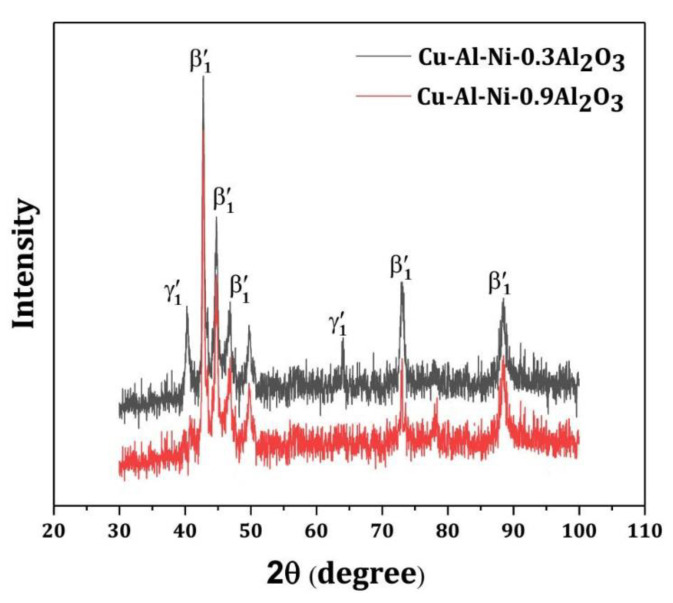
X-ray diffraction patterns of mid layers.

To precisely explore the phases in the mid layers, X-ray analysis was performed. It has been reported that the existence of the γ′_1_ phase falls in the range of around 40° and 65° [21]. Figure 7 confirms the co-existence of β′_1_ and γ′_1_ phases in Cu–Al–Ni-0.3Al_2_O_3_, whereas less possibility of a γ′_1_ phase was detected in Cu–Al–Ni-0.9Al_2_O_3_.

### 3.2. Mechanical Properties

**Figure 8:**
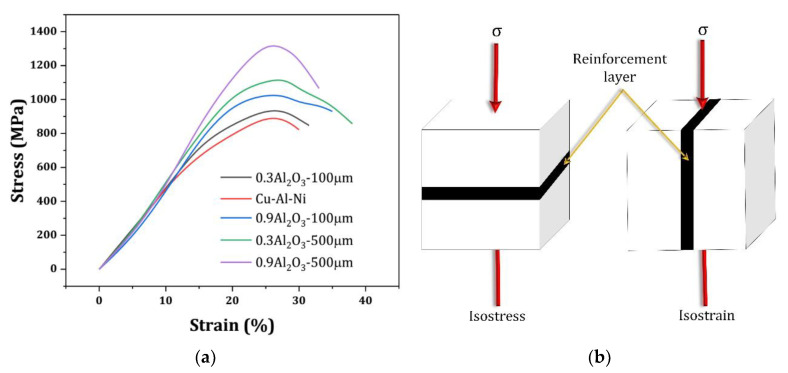
(**a**) The compressive plots. (**b**) Schematic of isostress and isostrain composites.

At the temperature range of around 25 °C (room temperature), compression tests were carried out to examine the mechanical properties and the SME of the samples built using two different reinforcement layer thicknesses (100 and 500 µm). The compressive stress–strain curves are shown in Figure 8a. The samples show similar elastic moduli but different compressive fracture strengths and flow behaviors. The plot indicates that at a lower reinforcement thickness, the sample with higher reinforcement content underwent a higher fracture strain, whereas at a higher reinforcement layer thickness, the sample with lower reinforcement content exhibited a higher fracture strain. The effects of these observations on the SME are briefly discussed. In Cu–Al–Ni-0.9Al_2_O_3_, the variant refinement shown in Figure 6b might be responsible for its high fracture strength.

The reason for the similar trend in the elastic modulus of samples might be related to the “inverse rule of mixtures”. In a composite material with a certain arrangement of reinforcement layers, the material property is a function of the loading direction. It is then known as an anisotropic composite, i.e., its strength and stiffness have different values in different directions. If the reinforcement layer is perpendicular to the loading direction, it is an isostress composite [22]. The loading direction significantly influenced the mechanical behavior of the reinforced composite. Considering the isostrain and isostress models depicted in Figure 8b, the elastic modulus is different for each condition. In this study, the positioning of the reinforcement layer was assumed to be similar to that in the isostress condition. Thus, the stress in the composite, ɓ_C_, is equal to the matrix stress, ɓ_M_, and reinforcement stress, ɓ_r_:

ɓ_C_ = ɓ_M_ = ɓ_r_ (1)

The composite deformation, ∆L_C_, in the direction of loading is the sum of the matrix and reinforcement deformation and comes from the strain, ε_C_, and thickness or volume, V. Thus, we have

∆L_C_ = ∆L_M_ + ∆L_r_ (2)

ε_C_ = V_M_ ε_M_ + V_r_ ε_r_ (3)

Assuming elastic behavior, the strain can be expressed in terms of stress, ε = ɓ/E. From Equation (1), the inverse rule of mixtures can be written as follows:

(4) $$\frac{1}{E_{C}}=\frac{V_{M}}{E_{M}}+\frac{V_{r}}{E_{r}}$$

This equation implies that the increase in the composite modulus under isostress is not significantly affected by the low reinforcement content (V_r_). Therefore, this study sheds new light on the demand for the further investigation of the orientations of local reinforcement layers in the 3D printing of metallic materials for future studies.

**Figure 9:**
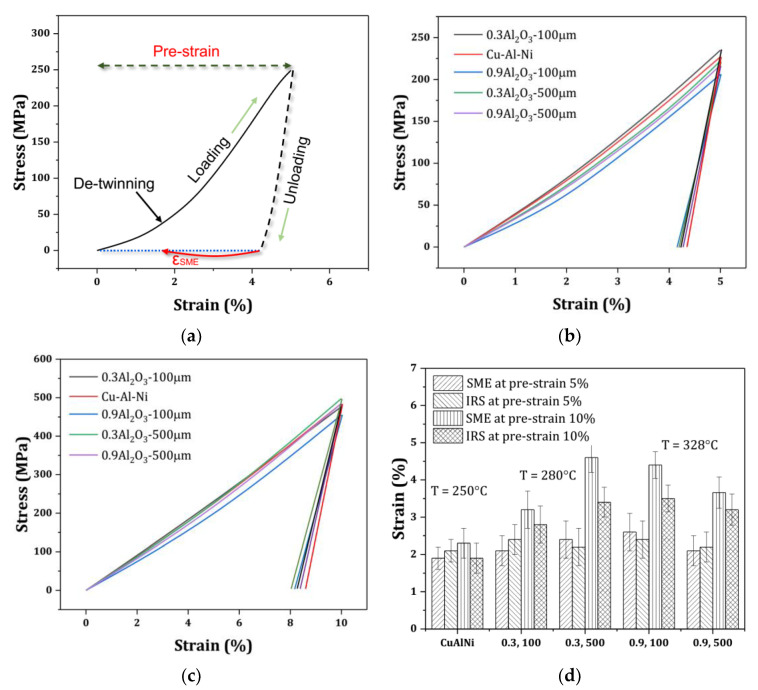
(**a**) Visualization of pre-strain and SME. Blue dashed line denotes a full recovery. Stress–strain curves were obtained under the pre-strains of (**b**) 5% and (**c**) 10%. (**d**) Quantitative values of the shape memory effect (SME) and irrecoverable strain (IRS) obtained under 5 and 10% pre-strain. Here, 0.3 and 0.9 denote reinforcement content, and 100 and 500 denote the layer thickness of the reinforcement layer. T: the temperature at which the sample is heated to obtain SME.

Figure 9a shows a typical stress–strain plot with the visualization of the pre-strain and ε_SME_. The plots have variations in their slope lines along the flow direction. A reduction in the early stages might imply a partial detwinning of the variants, similar to a stress-plateau after an insignificant elastic deformation that is followed by elastic deformation. This is frequently observed in the compression test because the detwinning anisotropy of the martensite in the compression test is different from that in the tension test. Less recovery emerges from compression due to the limited and partial detwinning, which is a result of heavy martensite collision or dislocation generation via compression [15]. This leads to a low partial detwinning at the beginning of the material flow and low shape recovery.

As stated previously, ε_SME_ was equal to (L_2_ − L_1_)/L_0_ × 100 when the height of the sample after unloading was L_1_, and the height was L_2_ when the sample fell to room temperature from Ap (austenite average temperature), where L_0_ denotes the initial height of the sample. Figure 9b,c illustrates the stress–strain curves under 5 and 10% pre-strain with quantitative values exhibited in Figure 9d that show the SME and irrecoverable strain (IRS) of the samples. At 5% pre-strain, the values did not show remarkable fluctuations in SME, which might be due to the small difference in the elastic modulus deduced from the compressive plots. According to this figure, with an increase in pre-strain to 10%, an increase in SME is evident in the samples. The difference between SME and IRS was also increased, which is beneficial for damping applications. The increased SME may be a result of the enhanced mismatch between the reinforcement particles and the matrix around the neutral plane. When a sample is under compression, it is plausible that the additive hard element is under compression, whereas the entire matrix is under tension [22,23]. Thus, the increase in pre-strain augments the stress mismatch between the reinforcement and the matrix around the neutral plane, which plays an important role in storing energy [24]. This improved the SME.

**Figure 10:**
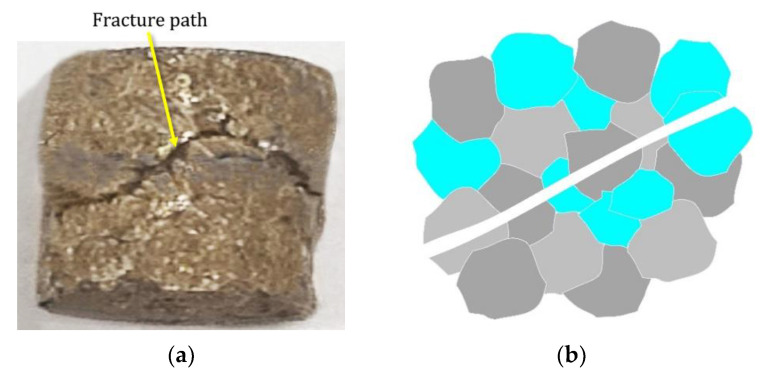

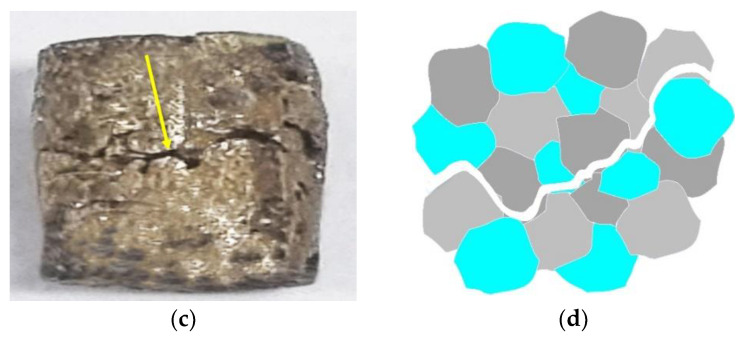
(**a**) Macroscopic image of fracture under 0.3 wt% Al_2_O_3_ and t = 500 µm. (**b**) Schematic of transgranular fracture. (**c**) Fracture in Cu–Al–Ni alloy. (**d**) Schematic of intergranular fracture. For clarity of the fracture mode, various colors are assigned to the grains.

**Figure 11:**
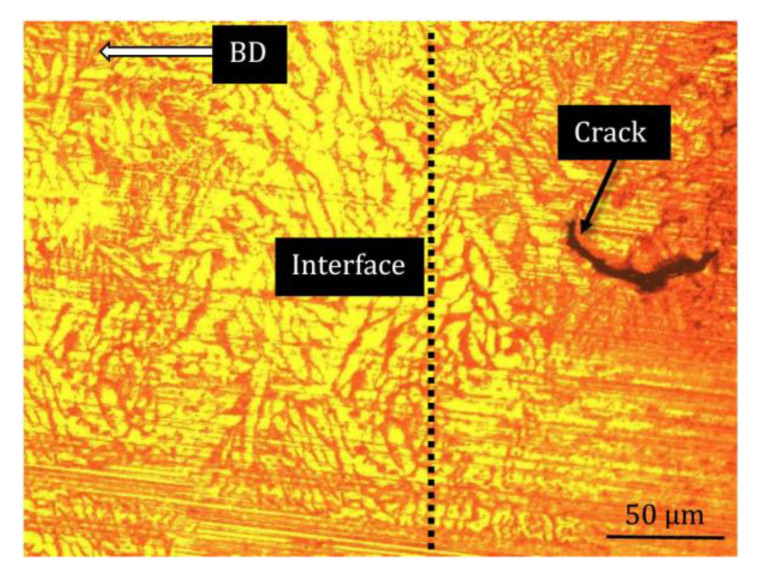
Defect in Cu–Al–Ni-0.9Al_2_O_3_ with t = 500 µm.

The higher SME is observed at the lower thickness of the reinforcement layer (t) when the reinforcement content is higher, i.e., 0.9 wt%. The SME was high at a high reinforcement layer thickness when the reinforcement content was lower, i.e., 0.3 wt%. According to the sample behavior in the elastic region, it was deduced that the fracture mode and ultimate compressive strain were able to influence the SME [25]. In the current work, the fracture mode was examined using a rough estimation deduced from the macrosamples because a clear difference between the fracture paths was observed. The reason for higher SME could be related to either the transgranular fracture mode or higher fracture strain under t = 500 µm with 0.3 wt% of Al_2_O_3_ or t = 100 µm with 0.9 wt% of Al_2_O_3_. In contrast to the intergranular brittle fracture, which occurs when a brittle fracture passes through the grain boundaries [26], the transgranular fracture occurs when high stress intensity exists during loading [20], because the local reinforcement layer generates local stress accumulation in the built part that is able to create inhomogeneous behavior within the sample [9,27,28]. The propagation of the load in this case moves through the grains and does not cause a sharp fracture, as intergranular fractures do. As shown in Figure 10a, with a schematic of the transgranular fracture shown in Figure 10b, the crack likely followed a path along the fracture line during loading, similarly to the sine curve that the locally reinforced layer made by perverting the sharp propagation of the fracture path observed for the original Cu–Al–Ni alloy in Figure 10c. These abovementioned samples, 0.3 wt% Al_2_O_3_, t = 500 µm and 0.9 wt% Al_2_O_3_, t = 100 µm, showed better ductility in Figure 8a. According to Figure 10c, an intergranular fracture, which is representative of a brittle fracture at triple junctions, occurred in the original Cu–Al–Ni alloy. A schematic of this process is shown in Figure 9d. The sample of 0.9 wt% Al_2_O_3_ with the reinforcement layer of 500 µm showed better ductility than the original Cu–Al–Ni alloy in Figure 8a. Because the superelastic and shape memory properties of SMAs are influenced by the number of martensite variants [29,30], the lower SME of this sample was due to the smaller martensite variants at the reinforcement layer, as observed in Figure 6b. This is able to enhance martensite stabilization [20], which results in an extreme mismatch and, hence, the occurrence of defects at the interface in the deformed sample, as shown in Figure 11.

The extreme mismatch at the interface generates a stretched interlayer, which causes the formation of cracks near the interface, as shown in Figure 11. These defects were promoted under a high pre-strain of 10%. Thus, the optimized values of the reinforcement layer and reinforcement content must be considered when the material is locally reinforced.

The findings of this study demonstrated that local reinforcement techniques could increase the SME of the Cu–Al–Ni alloy by 4.5% rather than requiring more reinforcement for the entire matrix. As was previously demonstrated [9], the fully reinforced sample did not show a remarkable improvement in SME. This was due to the hard-to-achieve uniform distribution of particles inside the printed part and, hence, the intricate anisotropy of stress generated within the part instead of the generation of a programmed stress anisotropy.

## 4. Conclusions

To increase the SME of Cu–Al–Ni SMA, a method that uses a reinforcement element that is not fully distributed within the matrix is required. The reinforcement particles were mixed with the matrix, and the mixture was deposited at the center of the 3D-printed samples in an area around the neutral plane. Two different percentages of alumina were employed as reinforcement agents to deposit the mixture at two different thicknesses. The selected values are representative of the low and high values, respectively. The key findings are as follows:

1. The reinforcement particles can break martensite variants rather than grains. With an increase in Al_2_O_3_ content, the martensite variants were continuously refined, which is deleterious to SME.
2. The compressive samples show almost similar elastic moduli, but different compressive fracture strengths and flowing behaviors led to different SME behaviors.
3. With the increase in pre-strain from 5% to 10%, the SME was increased, and an increase in the SME was observed. The increased SME was a result of the enhanced mismatch between the reinforcement particles and matrix around the neutral plane.
4. A higher SME was achieved at a lower thickness of the local reinforcement layer accompanied by higher reinforcement content. The fracture mode and higher fracture strain are responsible for this observation.
5. The sharp propagation of the fracture path in the original Cu–Al–Ni alloy was inhibited by the locally reinforced layer, showing a curved fracture path. This implied the better ductility of the material, accompanied by a higher fracture strain, indicating an improvement in the brittle fracture at the triple junctions of the Cu–Al–Ni SMA.

## Figures and Tables

**Table 1:** DSC results.

ID	M_s_ (°C)	M_f_ (°C)	A_s_ (°C)	A_f_ (°C)
Cu–Al–Ni	184	75	223	275
Cu–Al–Ni-0.3Al_2_O_3_	225	103	246	320
Cu–Al–Ni-0.9Al_2_O_3_	340	255	293	364

**Table 2:** The EDS analysis results of numbering points in Figure 5b,c.

Spectrum	Cu	Al	Ni	O
1	81.0	11.5	7.4	0.1
2	82.7	12.2	5.0	0.1

## Data Availability

Not applicable.
